# Vitamin B12 deficiency in metformin-treated community-dwelling older adults with diabetes: A cross-sectional multicentre study in Perak

**DOI:** 10.51866/oa.718

**Published:** 2025-12-22

**Authors:** Kin Wei Chua, Abdul Rani Rosilawati, Xing Yi Tang, Ahmad Tajuddin Ainul Hana, Sivaraja Yogeswary, Sariban Suriyati, Razali Norwani, Zainal Abidin Sofiah, Chin Aun Liew, Wee Kooi Cheah

**Affiliations:** 1 BPharm, MScMed(ClinEpi), Clinical Research Centre, Hospital Taiping, Taiping, Perak, Malaysia. Email: chuakinwei@crc.moh.gov.my; 2 MD, MPH, Clinical Research Centre, Hospital Taiping, Taiping, Perak, Malaysia.; 3 MBBS, Clinical Research Centre, Hospital Taiping, Taiping, Perak, Malaysia.; 4 M.D., M.Path.(Chemical Pathology), Department of Pathology, Hospital Taiping, Taiping, Perak, Malaysia.; 5 B.Biomedical.Sc., M.Sc(Management), Jabatan Patologi, Hospital Taiping, Taiping, Perak, Malaysia.; 6 MBBS, M.MED (Family Medicine), Klinik Kesihatan Bagan Serai, Bagan Serai, Perak, Malaysia.; 7 MBBS, Klinik Kesihatan Simpang, Taiping, Perak, Malaysia.; 8 MD, MMed(Family Medicine), Klinik Kesihatan Padang Rengas, Padang Rengas, Perak, Malaysia.; 9 MBBS, MRCP, Department of Internal Medicine, Hospital Taiping, Taiping, Perak, Malaysia.; 10 MBBS, MRCP, Department of Medicine and Clinic Research Center, Hospital Taiping, Taiping, Perak, Malaysia.

**Keywords:** Aged, Diabetes mellitus, Vitamin B12 deficiency, Metformin

## Abstract

**Introduction:**

Surveillance studies have shown a high prevalence of vitamin B12 deficiency in metformin-treated adults. Community-dwelling older adults have a higher risk of vitamin B12 deficiency due to lower gut absorption. To date, data on this specific cohort are lacking. We aimed to investigate the local prevalence of vitamin B12 deficiency and its associated factors among metformin-treated older adults.

**Methods:**

This prospective cross-sectional study investigated community-dwelling patients with diabetes aged ≥65 years from four healthcare facilities from August 2023 to March 2024. Blood samples were sent to a central laboratory to determine vitamin B12 levels. Vitamin B12 deficiency was defined as serum vitamin B12 levels of ≤221 pmol/L. Descriptive analysis, chi-square test (or Fisher’s exact test) and independent t-test (or Mann-Whitney U test) were performed.

**Results:**

One hundred two participants were included in the study, of whom 61.8% were women (n=63), and 55.9% were Malays (n=57). The median age was 70 years (interquartile range [IQR]=67-74), and the median duration of metformin usage was 9.0 years (IQR=5.0-12.3). The prevalence of vitamin B12 deficiency was 29.4% (95% confidence interval [CI]=20.4-38.4, n=30), with a median vitamin B12 level of 287.0 pmol/L (IQR=200.5-448.8). Vitamin B12 deficiency was significantly associated with advancing age (P<0.05), but not with sex, ethnicity, duration of diabetes, metformin use, BMI, latest HbA1c level recorded and haemoglobin level (P>0.05).

**Conclusion:**

Patients with diabetes aged ≥65 years have a high prevalence of vitamin B12 deficiency. Blood screening for vitamin B12 deficiency is needed for this population in Peninsular Malaysia.

## Introduction

Type 2 diabetes mellitus (T2DM) is a common illness among older adults. The estimated prevalence of diabetes mellitus is 28% in individuals aged 60 years and older in Malaysia, according to the National Health and Morbidity Survey 2018.^1^ Metformin is recommended as the first-line treatment for T2DM in the Malaysian clinical practice guidelines.^2^ This drug interferes with vitamin B12 absorption, particularly in long-term users. This interference is mediated by several mechanisms that contribute to the malabsorption of cobalamin (vitamin B12),^3,4^ especially in older adults, who are already at risk due to age-related physiological changes.^5,6^

Vitamin B12 or cobalamin malabsorption and deficiency due to inadequate dietary intake are quite common in older adults.^5^ They also have an increased risk of pernicious anaemia due to their advanced age.^6^ Studies have shown a prevalence of 5%-40% of vitamin B12 deficiency among free-living older adults, depending on the cutoff points used for blood vitamin B12 levels.^7-9^ Additionally, long-term metformin treatment may increase the risk of vitamin B12 deficiency.^10^ Metformin is the most common medical pill used in patients with diabetes mellitus, prescribed to about 84% of them.^11^ Moreover, the National Pharmaceutical Regulatory Agency has raised that healthcare professionals should be vigilant about the risk of vitamin B12 deficiency with metformin usage.^12^

In Malaysia, the prevalence of vitamin B12 deficiency among adults on metformin is approximately 28%.^13^ The risk of vitamin B12 deficiency is estimated to be higher in older adults with long-term use of metformin,^14^ where metformin induces the malabsorption of vitamin B12.^5^

Neuropsychiatric manifestations may occur in the absence of haematological abnormalities due to vitamin B12 deficiency.^15,16^ Some symptoms include paraesthesia, weakness, gait abnormalities and cognitive or behavioural changes. Furthermore, vitamin B12 deficiency can occur without anaemia or macrocytosis. The prevalence ranges from 5% to 10% for blood vitamin B12 levels of <140 pmol/L among patients without macrocytosis.^18^

Vitamin B12 is an essential vitamin. The human body is unable to produce vitamin B12; hence, obtaining sufficient amounts of vitamin B12 from dietary intake is crucial. The natural sources of vitamin B12 come from animal foods such as meat, fish, milk, cheese and eggs. Foods fortified with vitamin B12 or vitamin B12 supplements are other sources of dietary intake of vitamin B12. Plant-based foods do not naturally contain vitamin B12. Therefore, vegetarians are at risk for developing vitamin B12 deficiency owing to suboptimal intake. The reported prevalence of vitamin deficiency among vegetarians is high, reaching 11%—90% among older adults.^19^

Older adult patients with T2DM on metformin should be screened for blood vitamin B12 levels annually. However, a randomised controlled trial showed that adults more than 75 years old on metformin were less likely to be tested for vitamin B12 deficiency in primary care.^20^ Our study aimed to investigate the burden of vitamin B12 deficiency among older adults aged 65 years and above with diabetes on metformin to emphasise the need for routine screening for this group; it is essential to make a prompt and cost-effective diagnosis to prevent vitamin B12 deficiency complications in this population, especially irreversible neurological and/or neuropsychiatric disorders among older adults.

## Methods

This prospective cross-sectional study was conducted among older adult patients aged 65 years and above with diabetes from four healthcare facilities from August 2023 to March 2024. A convenience sampling method was adopted for the recruitment of participants. Vitamin B12 deficiency was defined as a serum vitamin B12 level of <221 pmol/L.^13,21^

The study population consisted of older adult patients with T2DM who attended the outpatient departments of the selected healthcare facilities. The inclusion criteria were as follows: age of 65-100 years, long-term use (minimum of 6 months) of metformin with a current metformin dose of at least 1500 mg/day, regular outpatient follow-up and provision of written informed consent. The exclusion criteria included the following: a history of gastric bypass, partial or complete gastrectomy, gastric reduction, bariatric surgery or chronic gastritis due to *Helicobacter pylori* infection; long-term use (>12 months) of medications affecting gastric acid secretion or pH (e.g. PPIs, H2RAs or antacids); diagnosis of Crohn’s disease, celiac disease or pernicious anaemia; vegetarian diet; vitamin B12 supplementation or injection within the past 3 months; presence of any severe illness (e.g. sepsis, malignancy, cirrhosis, heart failure or renal failure); alcohol abuse; and hospitalisation within the last 3 months.

Informed consent was obtained from each participant. Demographic characteristics and clinical histories were collected through patient interviews and review of clinical records. A total of 6 mL of venous blood was drawn from each participant to perform a full blood count (FBC) and measure serum vitamin B12 levels.^22,23^ FBC was processed at each healthcare facility, while all samples were transported to a central laboratory. Serum vitamin B12 levels were measured using a chemiluminescent microparticle intrinsic factor assay (Alinity B12 reagent kit, 07P67).

Data analysis was conducted using SPSS version 25 (IBM Corp, Armonk, New York, United States). Descriptive data were expressed as means ± standard deviations for normally distributed continuous variables and as medians and interquartile ranges for skewed data. An independent t-test was used to compare normally distributed continuous variables. For skewed data, the Mann-Whitney U test was applied. Categorical data were analysed using the chi-square test or Fisher’s exact test, as appropriate. A P-value of less than 0.05 was considered statistically significant.

### Post-hoc power analysis

A post-hoc power analysis was conducted to evaluate the adequacy of the sample size. For categorical variables (e.g. vitamin B12 level by age), the chi-square test assuming a moderate effect size (Cohen’s h=0.5) and an a-value of 0.05 yielded a post-hoc power of 99.9%, indicating sufficient power for detecting group-level differences. However, for continuous variables such as metformin usage duration and age, the independent samples t-tests assuming small-to-moderate effect sizes (Cohen’s d=0.3 for metformin usage duration and 0.35 for age) yielded post-hoc powers of 27.7% and 35.8%, respectively. These results suggest that the study may have been underpowered to detect small differences in some subgroup analyses.

## Results

### Patient characteristics

Within the study period of 8 months (August 2023 to March 2024), 102 participants were recruited. Table 1 shows the overview of the demographic and clinical characteristics at the presentation of the participants. The majority of the participants were aged 65-74 years (78%), women (62%) and Malays (56%). All participants were taking metformin, with a total dose of 1500 mg or 2000 mg/day, while none were current alcohol drinkers.

**Table 1:** Baseline demographic and clinical characteristics of the participants.

Characteristic	Total (N=102)
**Age, n (%)**	
65-74 years	80 (78.4)
75-84 years	22 (21.6)
**Sex, n (%)**	
Female	63 (61.8)
Male	39 (38.2)
**Ethnicity, n (%)**	
Malay	57 (55.9)
Chinese	22 (21.6)
Indian	23 (22.5)
BMI, median (IQR)	25.7 (23.1-29.6)
Current smoker, n (%)	4 (3.9)
Current alcohol drinker, n (%)	0
Duration of DM (year), median (IQR)	10.0 (6.0-13.0)
Duration of metformin usage (year), median (IQR)	9.0 (5.0-12.0)
Presence of neuropathy, n (%)	18 (17.6)
Latest HbA1c level (%), median (IQR)	7.4 (6.7-9.1)
Haemoglobin level (g/dL), mean (SD)	12.91 (1.672)
MCV (fL), mean (SD)	87.31 (5.919)
MCH (pg), median (IQR)	28.7 (27.5-30.5)
TWC (10^9/L), median (IQR)	8.4 (7.3-9.7)
Platelet count (10^9/L), mean (SD)	280.2 (75.22)
Vitamin B12 level (pmol/L), median (IQR)	287 (201-449)

**IQR=interquartile range; SD=standard deviation.

### Vitamin B12 deficiency

**Figure 1:**
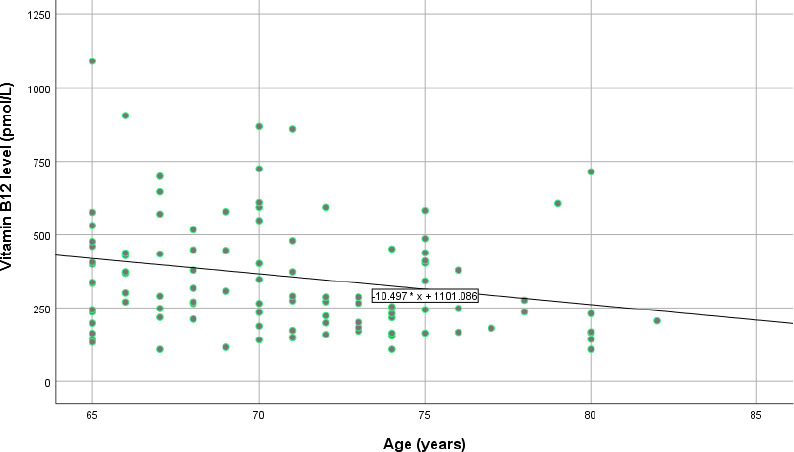
Scatter diagram of serum vitamin B12 level against age.

Among the 102 participants, 30 were found to have vitamin B12 deficiency (29.4%, 95% CI=20.4-38.4). A comparison of the clinical characteristics between the two study groups (normal vitamin B12 group versus vitamin B12 deficiency group) is presented in Table 2. Other characteristics such as sex, body mass index (BMI), duration of diabetes mellitus, metformin use, HbA1c level, haemoglobin level and mean corpuscular volume (MCV) did not show any significant difference between the groups. An exception was noted in the older age category in the vitamin B12 deficiency group (P=0.033), indicating that the participants with vitamin B12 deficiency were slightly older. Moreover, a weak difference was noted in the longer duration of metformin use in the vitamin B12 deficiency group (P<0.1). Figure 1 shows a scatter diagram of the serum vitamin B12 levels against the participants’ age, with a correlation coefficient of -0.215, indicating a weak negative correlation.

**Table 2:** Comparison of the clinical characteristics between the participants with and without vitamin B12 deficiency.

Characteristic	Normal vitamin B12 (n=72)	Vitamin B12 deficiency (n=30)	P-value
Age, median (IQR)	70 (66-74)	73 (69-75)	0.033
**<2 Ç/5**			
Female	45 (71.4)	18 (60.0)	0.813
Male	27 (28.6)	12 (40.0)	
**Ethnicity, n (%)**			
Malay	43 (75.4)	14 (46.7)	0.357
Chinese	13 (59.1)	9 (30.0)	
Indian	16 (69.6)	7(23.3)	
BMI, median (IQR)	25.0 (23.2-29.6)	26.5 (22.5-30.5)	0.615
Presence of neuropathy, n (%)	14 (19.4)	4(13.3)	0.461
Duration of DM (year), median (IQR)	10 (6-12)	10 (8-15)	0.138
Durationof metformin use (year), median (IQR)	8(5-11)	10 (6-14)	0.096
Latest HbAlc level, median (IQR)	7.5 (6.7-9.4)	7.1 (6.5-8.6)	0.313
Haemoglobin level (g/dL), mean (SD)	12.9 (1.56)	12.9 (1.94)	0.928
MCV (fL), mean (SD)	87.5 (5.53)	86.8 (6.84)	0.611
MCH (pgl, median (IQR)	28.7 (27.7-30.5)	29.1 (26.7-30.5)	0.929
TWC (10^9/L), median (IQR)	8.42 (7.55-9.51)	8.20 (6.65-11.45)	0.770
Platelet count (10^9/L), mean (SD)	277 (71.7)	287 (83.8)	0.552

### Concomitant medication use

Statins (n=66, 99.0%), ACEis/ARBs (n=76, 74.5%) and sulphonylureas (n=66, 61.7%) were the top three medicines consumed by the participants, while all were on metformin. All medications listed in Table 3 did not significantly differ between the groups.

**Table 3:** Concomitant medication use in the participants with and without vitamin B12 deficiency.

Medication	Total (N=102)	Normal vitamin B12 (n=72)	Vitamin B12 deficiency (n=30)	P-value
Sulphonylurea	66 (61.7)	49 (68.1)	17 (56.7)	0.273
Statin	101 (99.0)	71 (98.6)	30 (100)	1.000
DPP-IV antagonist	13 (12.7)	8 (11.3)	5 (16.7)	0.520
ACEi/ARB	76 (74.5)	54 (75.0)	22 (75.9)	0.928
CCB	20 (19.6)	12 (16.7)	8 (26.7)	0.246
Insulin	33 (32.4)	25 (35.7)	8 (27.6)	0.435
Beta-blocker	10 (9.8)	6 (8.3)	4 (13.3)	0.475
Aspirin	31 (30.4)	22 (30.6)	9 (30.0)	0.956

## Discussion

This study highlights the significant prevalence of vitamin B12 deficiency (29.4%) in older adult patients with diabetes on long-term metformin therapy, consistent with a previous report showing metformin’s negative impact on vitamin B12 absorption.^4^ The observed association between increasing age and vitamin B12 deficiency (P=0.033) aligns with prior findings indicating that advancing age exacerbates the risk of vitamin B12 deficiency due to physiological changes such as reduced gastric acid production and diminished intrinsic factor, both of which are critical for vitamin B12 absorption. This association is of particular concern in older adults, who may already be vulnerable to micronutrient deficiencies.^5,6^

### Age and vitamin B12 deficiency

The data suggest that older adult patients with diabetes, especially those over 65 years old, are at a higher risk of developing vitamin B12 deficiency, which can remain asymptomatic until serious complications arise. The significant inverse relationship between age and serum vitamin B12 levels emphasises the importance of routine monitoring, particularly for older patients on long-term metformin therapy. These findings support existing evidence that as individuals age, physiological barriers to proper vitamin B12 absorption increase, and this risk is compounded by chronic use of medications that further impair nutrient absorption such as metformin.^5,6^

### Interpretation of the weak correlation

The observed negative correlation between age and serum vitamin B12 levels (r=—0.215) was significant but weak. While not strongly predictive on its own, this modest inverse relationship supports existing literature suggesting that advancing age is associated with declining vitamin B12 levels.^5,6^ In older adult populations, even small shifts in micronutrient levels may reflect meaningful physiological changes due to cumulative exposure to age-related risk factors such as atrophic gastritis, reduced intrinsic factor and polypharmacy.

Notably, unmeasured confounders, such as dietary intake patterns, chronic gastrointestinal inflammation, undiagnosed malabsorptive conditions or genetic variants affecting vitamin B12 metabolism, could contribute to the weak correlation identified.^5,19^ Future studies should adopt a broader biopsychosocial framework, incorporating nutritional data, comorbidity indices and pharmacogenomics to more accurately elucidate the drivers of vitamin B12 deficiency in older adult populations with diabetes.

### Metformin and vitamin B12 absorption

Although no significant difference was found in the duration of metformin use between the normal vitamin B12 and vitamin B12 deficiency groups, the observed trend of longer metformin use in the latter aligns with previous data suggesting that prolonged metformin therapy interferes with vitamin B12 absorption.^4,10^ This interference occurs via disruption of calcium-dependent membrane action in the ileum, the primary site of vitamin B12 absorption.^3^ The fact that metformin is a first-line treatment for T2DM in Malaysia further supports the need for enhanced vigilance and regular monitoring of vitamin B12 levels in this population.^2^

### Neuropathy and clinical implications

Although neuropathy was not significantly associated with vitamin B12 deficiency in this study, the higher prevalence of neuropathy in the vitamin B12 deficiency group underscores the clinical relevance of early detection and intervention. Vitamin B12 deficiency is a known cause of peripheral neuropathy, which can be particularly debilitating for patients with diabetes.^15,16^ Neuropathy associated with vitamin B12 deficiency is often reversible with supplementation if detected early, but delayed diagnosis can lead to irreversible nerve damage and a significant decline in the quality of life. This study highlights the importance of integrating vitamin B12 monitoring into routine clinical care for older adult patients with diabetes to mitigate the potential risk of neuropathy and other neurological complications.

Public health implications and screening strategies The findings of this study underscore the need for targeted public health interventions, especially in Malaysia, where the older adult population is growing, and diabetes prevalence is increasing. Routine vitamin B12 screening for older adult patients with diabetes on long-term metformin therapy should be considered a standard part of outpatient care. Annual vitamin B12 level checks could allow for early detection of deficiency, preventing the onset of serious complications such as neuropathy, cognitive decline and anaemia.^6,20^ Such preventive measures could improve patient outcomes and reduce longterm healthcare costs by avoiding the treatment of advanced complications of vitamin B12 deficiency.

### Power and sample size considerations

The post-hoc power analysis demonstrated that while the study was sufficiently powered (99.9%) to detect moderate differences in the categorical variables, it was underpowered for detecting small-to-moderate differences in the continuous variables. Specifically, the post-hoc power for detecting differences in metformin usage duration and age was 27.7% and 35.8%, respectively. These findings highlight the potential for type II error, particularly in secondary analyses or subgroup comparisons involving continuous variables. Future studies with larger sample sizes are warranted to improve statistical power and enhance the ability to detect subtle but clinically relevant associations.^17^

### Interpretation of the non-significant findings

While several clinical variables including sex, BMI, HbA1c level, haemoglobin level and MCV did not demonstrate significant differences between the vitamin B12 deficiency and normal vitamin B12 groups, this does not necessarily indicate a lack of clinical relevance. The possibility of type II error due to limited statistical power, especially for detecting small-to-moderate effect sizes, must be considered. Moreover, vitamin B12 status is likely influenced by multifactorial elements such as genetic polymorphisms, dietary intake patterns, medication adherence and unmeasured comorbidities, which were not captured in the present dataset. Future studies with larger sample sizes, dietary assessments and more granular clinical data are warranted to identify subtle or indirect predictors of vitamin B12 deficiency in this population.

### Nutritional intake considerations

Vitamin B12 status is influenced not only by absorption-related factors such as age and medication use but also by dietary intake. Inadequate consumption of animal-based foods, which are primary sources of vitamin B12, may contribute to low serum levels, particularly among individuals with dietary restrictions. However, detailed dietary data were not collected in this study due to resource limitations and the time constraints of a multicentre design focused on clinical and biochemical assessments. Although patients with a vegetarian diet were excluded, we acknowledge that variability in non-vegetarian dietary patterns could still influence vitamin B12 status. Future studies should consider incorporating validated dietary assessments to provide a more comprehensive evaluation of nutritional contributors to vitamin B12 deficiency.

### Impact of past supplementation andgeneralisability

Although patients who had received vitamin B12 supplementation within the past 3 months were excluded, earlier supplementation (e.g. within 6-12 months) may have influenced the current serum vitamin B12 levels. This could have resulted in misclassification of some participants’ deficiency status. Due to reliance on medical records and self-reporting, such longterm supplementation data were not consistently available and thus could not be accounted for.

Furthermore, the exclusion of individuals with a vegetarian diet and gastrointestinal disorders (e.g. conditions requiring gastrectomy, Crohn’s disease or chronic gastritis) and those on longterm acid-suppressive therapy means that the study findings may not be generalisable to these higher-risk groups. These subpopulations may exhibit different prevalence rates and risk profiles for vitamin B12 deficiency. Therefore, while our results are applicable to community-dwelling older adult patients with diabetes on metformin in outpatient settings, caution is warranted when extending the conclusions to the excluded subgroups.

### Strengths and limitations

This study has several notable strengths. First, it focused specifically on a high-risk population - older adult patients with diabetes receiving longterm metformin therapy - thereby providing clinically relevant insights that are directly applicable to outpatient settings. Second, the study employed rigorous exclusion criteria to eliminate confounding factors that independently influence vitamin B12 absorption, such as gastric surgeries, chronic gastritis and long-term use of acid-reducing medications. This strengthened the internal validity of the findings. Additionally, the use of a validated chemiluminescent microparticle intrinsic factor assay for measuring serum vitamin B12 levels ensured accuracy and reliability in the biochemical assessments. The study also included a diverse participant pool, comprising various ethnic groups reflective of Malaysia’s multicultural population, which may support broader applicability of the findings. However, generalisability remains limited due to the use of convenience sampling and the study’s sample size.

Nevertheless, the study is not without limitations. One key limitation is the relatively small sample size of 102 participants, which limited the study’s power to detect small-to-moderate effect sizes in the continuous variables. While the study achieved sufficient power (99.9%) for the categorical analyses, the post-hoc powers for the continuous variables were only 27.7% (metformin use duration) and 35.8% (age), indicating an increased risk of type II error in detecting subtle differences. Furthermore, the cross-sectional study design precluded any causal inference between metformin use and vitamin B12 deficiency. Longitudinal studies would be necessary to explore the temporal relationship and progression of vitamin B12 deficiency in this population. Another limitation is the absence of dietary intake data, which could influence serum B12 levels and may confound the interpretation of deficiency prevalence. Finally, while the study involved multiple healthcare facilities, all sites were located within a specific region of Perak, Malaysia, which may limit the generalisability of the findings to other regions with different demographic or healthcare characteristics.

This study’s cross-sectional design inherently limited the ability to draw causal inferences regarding the relationship between metformin use, age and vitamin B12 deficiency. While associations were observed, particularly with advancing age, the temporal sequence and potential confounding factors cannot be fully addressed without longitudinal follow-up. Furthermore, the relatively low post-hoc powers of 27.7% (for metformin use duration) and 35.8% (for age) may have compromised the study’s capacity to detect subtle but potentially meaningful associations, especially for variables such as the duration of metformin use and neuropathy. Consequently, the findings should be interpreted with caution, and future studies with larger sample sizes and prospective designs are warranted to validate and expand upon these results.

## Conclusion

This study highlights the high prevalence of vitamin B12 deficiency among older adult patients with diabetes on long-term metformin therapy, with nearly one-third of the participants affected. The significant association between increasing age and vitamin B12 deficiency reinforces the need for routine vitamin B12 screening in older adult patients, particularly those over 65 years old, to prevent serious complications such as neuropathy and cognitive decline. Early detection and intervention through vitamin B12 supplementation can mitigate these risks and improve patient outcomes. Incorporating routine vitamin B12 screening into outpatient care for older adults with diabetes on metformin is a clinically sound strategy. Although it may potentially reduce long-term healthcare costs, a formal cost-effectiveness analysis was not conducted in this study and should be addressed in future research. By preventing vitamin B12 deficiency and its associated complications, such preventive measures could significantly reduce healthcare costs and enhance the quality of life for this vulnerable population.

While the study was sufficiently powered for detecting moderate categorical differences, its limited power for the continuous variables underscores the need for larger sample sizes in future research.

## Data sharing statement

De-identified data that support the findings of this study are available from the corresponding author upon reasonable request and subject to approval by the Ministry of Health Malaysia.

**How does this paper make a difference in general practice?**

- Older adults on metformin have a higher risk of decreased blood vitamin B12 levels as they approach advanced age.
- Screening of blood vitamin B12 levels for older adults with diabetes who are on long-term metformin is important in outpatient clinic settings.
